# Supplementary material for: Biochemical characterization and inhibition of thermolabile hemolysin from Vibrio parahaemolyticus by phenolic compounds
Source: PeerJ. 2021 Jan 6;9:e10506. doi: 10.7717/peerj.10506 (PMC7796666; doi:10.7717/peerj.10506)
Supplement: File S1 — All aligned sequences have the highly conserved GDSL domain (green), SGNH catalytic domain (light blue) and the blocks part of the phospholipase A2 domain (red). [file peerj-09-10506-s001.docx]

**Sequences alignment of TLHs from genus Vibrio**. All aligned sequences have the highly conserved GDSL domain (green), SGNH catalytic domain (light blue) and the blocks part of the phospholipase A2 domain (red).

***V. parahaemolyticus*** MMKKTITLLTA-LLPLASAVAEEPTLSPE--MVSASEVISTQENQTYTYVRCWYRTSYSK 57

***V. cholerae*** -MKKRLSILIAGLASLSVNAATEPWASPEAEVLSRAQIQQVQGKQTYTYVRCWYRPAATH 59

***V. Anguillarum*** --MKLMTLLMTTILPLSMAIAENTDTTSE--PVTAYGVMNTQNKQTYTYVRCWYRPAGNH 56

***V. vulnificus*** --MKKITILLGALLPFTSAVADEPALSPE--AITSAQVFSTQSKETYTYVRCWYRTGNSH 56

***V. alginolyticus*** -MKKTITLLTA-LLPLASAIAEEPTLSPA--MVSAAEVVSAQENQTYTYVRCWYRTSHSK 56

***V. diabolicus*** MMKKTITLLTA-LLPLASAVAEEPTLSPE--MVSASEVVSTQENQTYTYVRCWYRTSHSK 57

***V. harveyi*** -MNKTITLLSALLLPLSFAHAAEPTLSPE--MVSASQVRSAQAKQTYTYVRCWYRTSYSK 57

***V. campbellii*** -MNKTITLLSALLLPLNLAHAADSSEPSY--QLNASEVRSTQQKQTYTYVRCWYRTSYSH 57

* :::* : : * : :. : ..* ::********** . .:

***V. parahaemolyticus*** DDPATDWEWAKNEDGSYFTIDGYWWSSVSFKNMFYTNTSQNVIRQRCEATLDLANENADI 117

***V. cholerae*** DDPYTTWEWAKNADGSYYTIQGYWWSSIRQKNMFYTTVQPETLLERCEETLGVNHDFADI 119

***V. anguillarum*** DDPATDWEWALDEQGNDYTISGYWWSSVRFKNMFYTDTSQYEIKQRCEETLGVSHDSADI 116

***V. vulnificus*** DESATDWEWAENPDGSYFTIDGYWWSSVRLKNMFYTNTSQNVIKQRCEETLGVTHDAADI 116

***V. alginolyticus*** DDAATDWKWAKNQDGSDFTIDGYWWSSVSFKNMFYTNTSQNVIRQRCEETLDLANENADI 116

***V. diabolicus*** DDAATDWKWAKNQDGSDFTIDGYWWSSVSFKNMFYTNTSQNVIRQRCEETLDLANENADI 117

***V. harveyi*** DEPATDWEWAENPDGSYFTLDGYWWSSVSFKNMFYTDTPQSVIKQRCEQTLDLANENADI 117

***V. campbellii*** DDPETDWEWAENPDGSYFTIEGYWWNALSFKNMFYTNTSQSVIKQRCEQTLDLANENADI 117

*: * *:** : :*. :*:.****.:: ****** . : :*** **.: :: ***

- Block I -

***V. parahaemolyticus*** TFFAADNRFSYNHTIWSNDAAMQPDQINKVVALGDSLSDTGNIFNASQWRFPNPNSWFLG 177

***V. cholerae*** TYFAADHRFSYNHTIWSNDPEVQSNRISKVIAFGDSLSDTGNIFNASQWRFPNPDSWFLG 179

***V. anguillarum*** TYFAADNRFSYNHSIWTNDKQNAKPVINKMVTIGDSLSDTGNIFNASQWKFPNPNSWFLG 176

***V. vulnificus*** TYFAADNRWSYNHTIWTNDPVMQADQINKIVAFGDSLSDTGNIFNAAQWRFPNPDTWFLG 176

***V. alginolyticus*** TFFAADNRYSYNHTIWSNDAAMQPDQINKVVALGDSLSDTGNIFNASQWRFPNPNSWFLG 176

***V. diabolicus*** TFFAADNRYSYNHTIWSNDAAMQPDQINKVVALGDSLSDTGNIFNASQWRFPNPNSWFLG 177

***V. harveyi*** TFFAADNRFSYNHTIWSNDPVMQPDQINKVVALGDSLSDTGNIFNASQWRFPNPNSWFLG 177

***V. campbellii*** TYFAADNRWSYNHSIWSNDPVMQPDQINKVVALGDSLSDTGNIFNASQWRFPNPNSWFLG 177

*:****:*:****:**:** *.*::::*************:**:****::****

- Block II -

***V. parahaemolyticus*** HFSNGFVWTEYIAKAKNLPLYNWAVGGAAGENQYIALTGVGEQVSSYLTYAKLAKNYKPA 237

***V. cholerae*** HFSNGFVWTEYLAQGLNVPLYNWAVGGAAGRNQYVALTGVYEQVSSYLSYMRLAKNYQPE 239

***V. anguillarum*** HFSNGLVWTEYLAKMHNLPVYNWAIGGAAGRNQYIALTGVNDQVSSYLNYMTMAKNYQPK 236

***V. vulnificus*** HFSNGFVWTEYIAQAKKLPLYNWAVGGAAGSNQYVALTGVKDQVLSYLTYAKMAKNYKPE 236

***V. alginolyticus*** HFSNGFVWTEYVAKAKNLPLYNWAVGGAAGENQYIALTGVGEQVSSYLTYTKLAKNYNPA 236

***V. diabolicus*** HFSNGFVWTEYVAKAKNLPLYNWAVGGAAGENQYIALTGVGEQVSSYLTYTKLAKNYNPA 237

***V. harveyi*** HFSNGFVWTEYIAQAKNLPLYNWAVGGAAGENQYIALTGVGEQVSSYLAYAKLAKNYKPA 237

***V. campbellii*** HFSNGFVWTEYIAQAKNLPLYNWAVGGAAGENQYIALTGVGEQVSSYLAYMQLAKNYNPA 237

*****:*****:*: ::*:****:***** ***:***** :** *** * :****:*

- Block III -

***V. parahaemolyticus*** NTLFTLEFGLNDFMNYNRGVPEVKADYAEALIRLTDAGAKNFMLMTLPDATKAPQFKYST 297

***V. cholerae*** NSLFTLEFGLNDFMNYNRSLADVKADYSSALIRLIDAGAKNLVLMTLPDATRAPQFQYAT 299

***V. anguillarum*** NTLFTLEFGLNDFMNYNREVYEVKADFSSAMIRLVDSGAKNIILLTLPDATKAPQFKYST 296

***V. vulnificus*** NTLFTLEFGLNDFMNYNREVVDVKADFSTALIKLTDAGAKNIMLMTLPDATKAPQFKYST 296

***V. alginolyticus*** NTLFTLEFGLNDFMNYNRSVPEVKADYAEALIRLTDAGAKNFMLMTLPDATKAPQFKYST 296

***V. diabolicus*** NTLFTLEFGLNDFMNYNRSVPEVKADYAEALIRLTDAGAKNFILMTLPDATKAPQFKYST 297

***V. harveyi*** NTLFTLEFGLNDFMNYNRSVPEVKSDYAEALIKLTDAGAKNLLLMTLPDATRAPQFTYST 297

***V. campbellii*** NTLFTLEFGLNDFMNYNRSVPEVKADYSEALIKLSDAGARNFLLMTLPDATYAPQFKYSS 297

*:**************** : :**:*:: *:*:* *:**:*::*:****** **** *::

***V. parahaemolyticus*** QEEIDKIRAKVLEMNEFIKAQAMYYKAQGYNITLFDTHALFETLTSAPEEHGFVNASDPC 357

***V. cholerae*** QEQIDTVRSKIIGMNAFIREQARYYQMQGIRIVLFDAYTLFDSITTQPEQHGFANANSPC 359

***V. anguillarum*** AEEIAKVSAKITTFNAFIQQQALYYQMQGVNIILFDAHSLFENLTANPQQYGFENATDAC 356

***V. vulnificus*** QAEIEKVRAKIVEFNEFIKAQAAFYIIQGYNITLYDTHGLFEQLTQNPQQHGFVNASDAC 356

***V. alginolyticus*** QEEIETIRAKVLKMNEFIKAQAMYYKAQGYNIALFDTHALFEKLTSAPEEHGFVNASDPC 356

***V. diabolicus*** QEEIETIRAKVLKMNEFIKAQAMYYKAQGYNISLFDTHALFETLTSAPEEHGFVNASDPC 357

***V. harveyi*** QEEINKIRAKIVEMNEFIKAQAAYYTAQGYNVTLYDTHALFESLTANPEQHGFVNASQAC 357

***V. campbellii*** QEEIDTIRAKILEMNEFIKAQAAYYTAQGLNITLHDTHALFASLTANPEQHGFSNATEAC 357

:* .: :*: :* **: ** :* ** .: *.*:: ** :* *:::** **.. *

- Block V -

***V. parahaemolyticus*** LDINRSSSVDYMYTHALRSECAASGAEKFVFWDVTHPTTATHRYVAEKMLESSNNLAEYR 417

***V. cholerae*** LDIRRNSAADYLLSHSLSAECAKQGSDRFVFWEVTHPTTAIHHYLAEQILAT--EMAQFP 417

***V. anguillarum*** LNINRNSALDYLYRHSLTNDCAYHSSDKYVFWGVTHPTTAIHQLIANEISKN--SLASFQ 414

***V. vulnificus*** LNINRASSADYLYSHSLTNDCATHSSDKYVFWGVTHPTTAVHKYIAEKMLAPGAGMQRFN 416

***V. alginolyticus*** LDINRSSSVDYMYTHSLRSECAASGADKFVFWDVTHPTTATHRYVAEKMLESSNNLEEFR 416

***V. diabolicus*** LDINRSSSVDYMYTHSLRSECAASGADKFVFWDVTHPTTATHRYVAEKMLESSNNLEEFR 417

***V. harveyi*** QDINRSSSVDYLYHHALRSECASSGSDKFVFWDVTHPTTATHHYVAEKMLESTNQLSNHP 417

***V. campbellii*** QDINRSSAADYLYKHSLRSECASVGSDKFVFWDVTHPTTATHRYVAEDMLETTDQLSNHP 417

:*.* *: **: *:* :** .::::*** ******* *: :*:.: : .

***V. parahaemolyticus*** F- 418

***V. cholerae*** L- 418

***V. anguillarum*** FE 416

***V. vulnificus*** F- 417

***V. alginolyticus*** F- 417

***V. diabolicus*** F- 418

***V. harveyi*** F- 418

***V. campbellii*** F- 418
