# Supplementary figures and images for: Biochemical characterization and inhibition of thermolabile hemolysin from Vibrio parahaemolyticus by phenolic compounds
Source: PeerJ. 2021 Jan 6;9:e10506. doi: 10.7717/peerj.10506 (PMC7796666; doi:10.7717/peerj.10506)

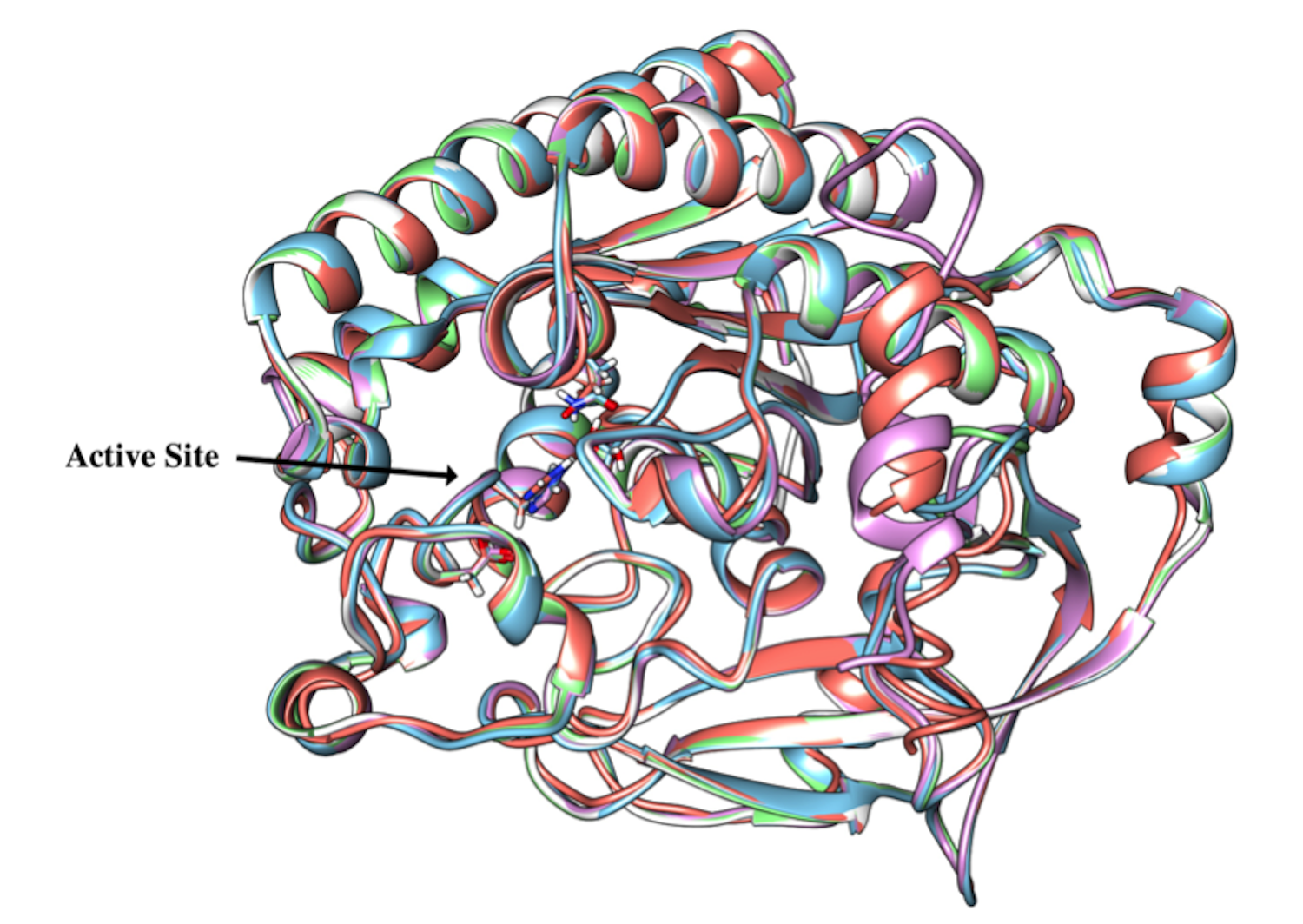

Supplement: File S2 — Algorithms used to obtain structural models were as follows: MOE (blue), SWISS-MODEL (green), Phyre2 (pink) and I-TASSER (orange) and 6JL1 (white). General RMSD: 0.384. The RMSD of predicted models using as reference the experimental structure of Vv TLH were 0.145 for SWISS-MODEL, 0.253 for Phyre2, 0.407 for MOE, and 0.335 for I-TASSER. [file peerj-09-10506-s002.png]

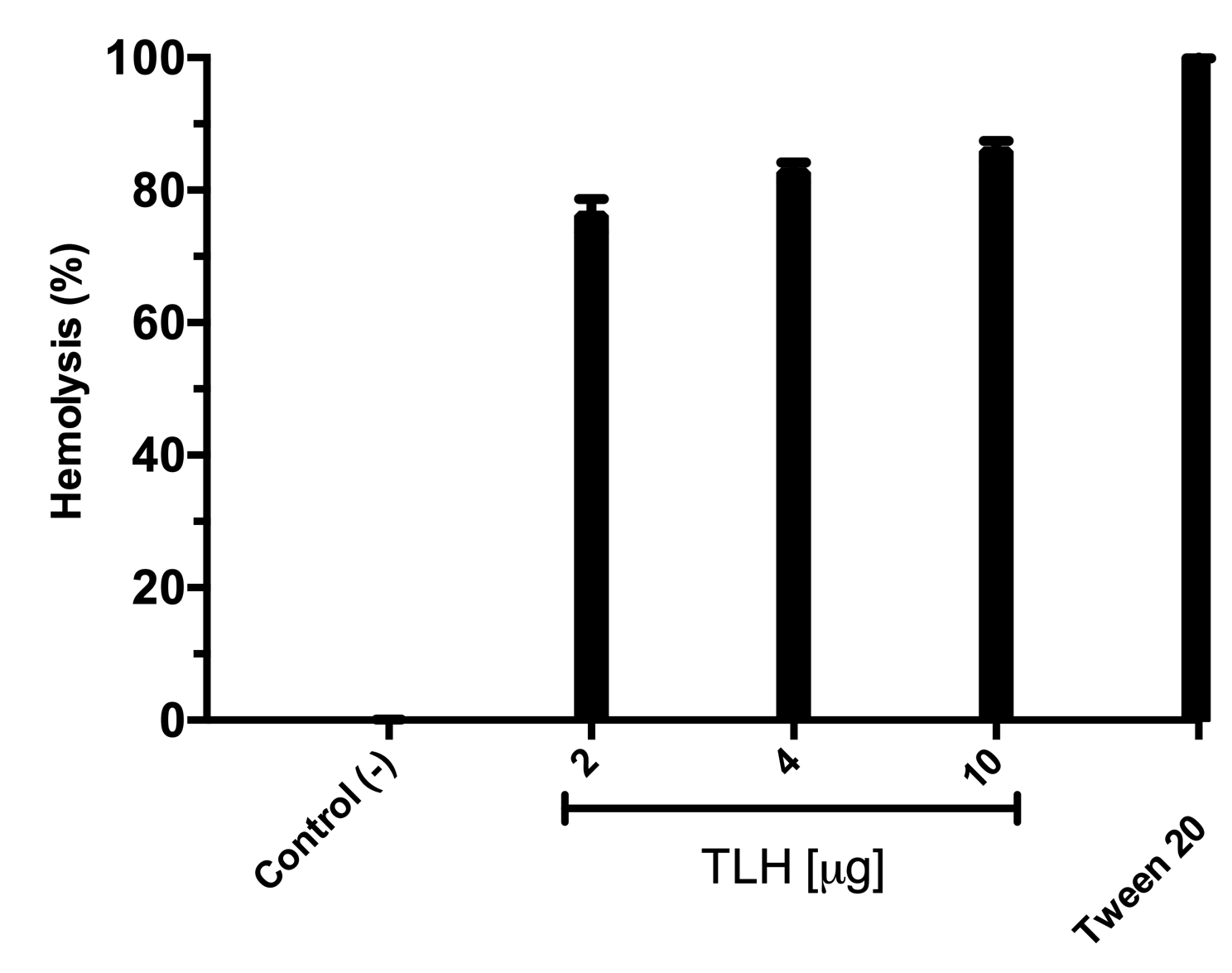

Supplement: File S3 — Tween-20 was considered as 100% hemolysis. Control (-), was buffer without in absence of TLH. [file peerj-09-10506-s003.png]
